# Prophylactic Treatment with Vedolizumab in the Prevention of Postoperative Recurrence (POR) in High-Risk Crohn’s Patients

**DOI:** 10.3390/jcm12093130

**Published:** 2023-04-26

**Authors:** Giuseppe Frieri, Marco Valvano, Sara Frassino, Susanna Faenza, Nicola Cesaro, Gianfranco Amicucci, Rosa Manetta, Angelo Viscido, Giovanni Latella

**Affiliations:** 1 Gastroenterology Unit, Division of Gastroenterology, Hepatology and Nutrition, Department of Life, Health and Environmental Sciences, University of L’Aquila, Piazzale Salvatore Tommasi 1, 67100 L’Aquila, Italyvalvano.marco@libero.it (M.V.);; 2 Department of Surgery, University of L’Aquila, 67100 L’Aquila, Italy; 3 Division of Radiology, S. Salvatore Hospital, 67100 L’Aquila, Italy

**Keywords:** Crohn Disease, inflammatory bowel disease (IBD), post operative recurrence (POR), Vedolizumab

## Abstract

About 50% of Crohn’s Disease (CD) patients undergo an intestinal resection during their lifetime. Although the patients experience a fairly long period of well-being after the intestinal resection, they presented a postoperative recurrence (POR) in 40% of cases within 5 years. In this case series, we aimed to evaluate the incidence of POR in CD patients with high risk for early POR, prophylactically treated with Vedolizumab. All consecutive CD patients (followed from 2017 to 2020) who underwent ileocolonic resection after the loss of response at anti-Tumor Necrosis Factor α (anti-TNFα) and with one or more risk factors for early POR were included. POR was defined as a Rutgeerts score (Ri) > 1 at the colonoscopic evaluation. All the included patients underwent a Magnetic resonance enterography (MRE) at least one year after the surgical resection. Six patients (4 Female; 2 Males) were included. At the first endoscopic evaluation, all patients were in endoscopic remission (5 patients Ri 0; 1 patient Ri 1). No stenosis nor other intestinal wall changes or complications were observed at MRE. Five patients underwent colonoscopy over two years of follow-up (median: 32 months; range 25–33). The Ri score was 0 in four patients, while the fifth patient showed severe endoscopic relapse. The same patient presented a clinical relapse (Harvey-Bradshaw index = 10) with a flare of disease in the colonic mucosa. These data suggest that early post-operative treatment with Vedolizumab could be a valuable strategy to be submitted to a prospective controlled trial for preventing POR.

## 1. Introduction

Crohn’s Disease (CD) is a chronic inflammatory disease that can affect any area of the gastrointestinal tract. Its natural course is characterized by periods of remission alternating with periods of flares, often controlled by drug escalation or a change of treatment strategy, this also includes surgery in 44.6% of cases within 10 years [1].

Historically, surgical resection was considered a failure of medical treatment and, therefore, something to avoid at any cost [1] despite a Randomized Controlled Trial (RCT) published in 2017 in which the authors showed that in CD patients with a non-structuring, ileocecal disease and failure with conventional therapy that the laparoscopic resection was a reasonable alternative to infliximab therapy [2]. Ileocecal resection represents the most frequent surgical intervention that assures a fairly long period of well-being interrupted by post operative recurrence (POR) in 40% of cases within 5 years and further resections in about one-third of them [3,4,5,6,7], the latter event being even more likely in high-risk patients [8].

Many strategies intended to prevent POR have been tested. Mesalazine, azathioprine, antibiotics, and anti-TNF have shown disappointing results. However, only prophylactic treatment with anti-TNF showed more significant results [9]. In fact, a few RCTs showed a lower rate of endoscopic and clinical recurrence in patients treated with anti-TNFα compared to azathioprine or mesalamine [10,11,12,13], and only a few retrospective studies compared anti-TNFα or Ustekinumab and Vedolizumab showing conflicting results [14,15,16].

A more promising approach to POR management was proposed by the randomized postoperative Crohn’s endoscopic recurrence trial (POCER). In this RCT, 122 out of 174 patients were enrolled in the active care group and modified their therapy according to the risk of recurrence based on the 6 months of colonoscopy, and 47 followed a step-up treatment strategy optimizing drug therapy alone. The author showed that at 18 months, 60 (49%) and 35 (67%) among the included patients presented a POR in the active care and in the standard care group, respectively (*p* = 0.03). Mucosal healing was maintained in 27 (22%) of 122 patients in the active care group versus 4 (8%) in the standard care group (*p* = 0.03) [17]. However, this is not a prevention strategy but rather an early treatment of an early endoscopic recurrence. The target of early treatment is achieving and maintaining mucosal healing, whilst the prevention target is the maintenance of the lesions’ absence, especially in high-risk patients. To date, a definitive long-term effective prevention strategy is not yet well established.

In this study, we report the results of a case series in which we treated a high-risk group of patients starting from the first post-operative weeks, strictly following them for an extended time, longer than reported so far. The need for this early preventive treatment was induced by the high probability of recurrence that characterizes these patients. To this purpose, we decided to use Vedolizumab, an anti-integrin drug that acts at an early stage of the inflammatory cascade on the assumption that this drug could prevent T lymphocytes from reaching the healthy peri-anastomotic mucosa of recently operated patients.

Vedolizumab, a humanized monoclonal antibody to the α4β7 integrin that selectively reduces intestinal lymphocyte trafficking [18] is considered an appropriate alternative in CD patients refractory to steroids and/or patients who have a primary non-response or secondary loss of response to anti-TNFα, according to the European guidelines (ECCO) [19,20].

## 2. Materials and Methods

This is a single-center observational study including all consecutive CD patients (from 2017 to 2020) who underwent ileocolonic resection after the loss of response at anti-TNFα and with one or more risk factors for early POR.

The diagnosis of CD was based on standard clinical, cross-sectional imaging techniques, endoscopic, and histological criteria [21].

All the patients were followed at the Gastroenterology, Hepatology, and Nutrition division of the University of L’Aquila. All clinical investigations followed the principles reported in the Declaration of Helsinki and according to the Strengthening the Reporting of Observational Studies in Epidemiology (STROBE) Statement guidelines [22]. Ethics approval was issued by the Internal Review Board of the University of L’Aquila (protocol number 11/2021). All subjects gave their informed consent for the current study.

### Procedures

All included patients started prophylactic therapy with a standard induction of Vedolizumab (300 mg at day 1 and weeks 2 and 6) and every 8 weeks, within 12 weeks from the ileocecal resection.

A colonoscopy with biopsies was performed after one year and over two years after surgery if the patients were in clinical remissions, or before, based on clinical needs. In case of inadequate bowel preparation (Boston scale < 6 or <2 in any of the colonic tracts) we planned to repeat the colonoscopy [23].

The histological activity was defined by the presence of inflammatory cell infiltrate in at least one of the histological sections [24].

POR was defined as a Rutgeerts score (Ri) > 1 at the colonoscopic evaluation [25].

Magnetic resonance enterography (MRE) was performed at least after one year following the surgical resection.

Clinical recurrence was evaluated using the Harvey-Bradshaw index (HBI). A score of ≥ 5 points identified a clinical recurrence. The clinical evaluation was carried out at each scheduled visit for the Vedolizumab maintenance therapy [25].

## 3. Results

Six patients (4 Female; 2 Male) who underwent an ileocecal resection for CD after the loss of response at anti-TNFα (4 for stenosis and 2 for stenosis and fistulation) were included.

These patients started prophylactic therapy with Vedolizumab (standard dose) after a median of 7 weeks (range: 4–12) after surgery. One patient also had a left colonic localization not surgically treated. The baseline characteristics and clinical data are reported in Table 1.

Four patients presented an ileocolonic disease (L3 Montreal classification) and two patients had a selective ileal involvement (L1 Montreal classification). All the patients included in our study had a high risk of POR and presented one or more predictors of early postoperative recurrence. The risk factors for an early POR are shown in Table 2.

### 3.1. Clinical Evaluation and Disease Activity at the End of Follow Up

During the observational period, only one patient presented a severe clinical relapse (HBI 10) after 33 months of follow-up. Despite the clinical remission, a relevant increase in C-reactive protein (CRP) was observed after 20 months (3.3 mg/dL). Infective diseases were ruled out and a complete biochemical analysis was performed. Considering clinical and endoscopic flare, she underwent a cycle of systemic steroids and a swop to Ustekinumab.

All the other patients were in clinical remission until the end of the observational period. Two patients presented a slight increase in CRP (0.8 mg/dL and 0.1 mg/dL in patients number 2 and 6, respectively). Moreover, none of the included patients underwent steroid treatment during the follow-up (Table 1). 

### 3.2. Endoscopic and Histological Evaluation

All of the included patients performed a colonoscopy with multiple biopsy sampling during the first year of follow-up (median 9 months; range: 8–12) and all were in endoscopic remission. Only one male patient presented two aphthous ulcers in the neo-distal ileum (Rutgeerts score: i1).

**Figure 1:**
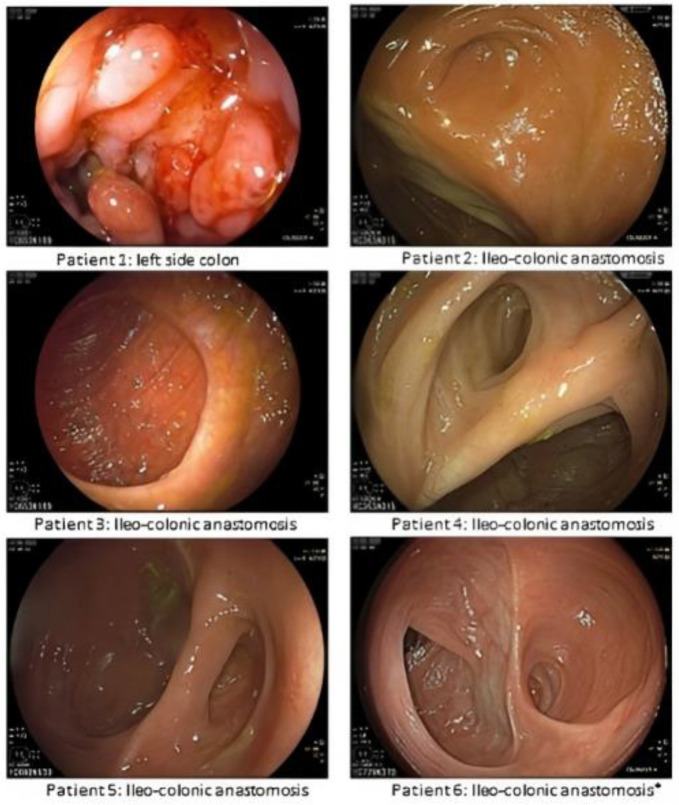
Second colonoscopic evaluation during the follow-up period. * First endoscopic evaluation.

Five patients underwent a second endoscopic evaluation with multiple biopsies during the follow-up period (median 32; range 25–33 months); 4/5 were in endoscopic remission (3 Rutgeerts 0 and 1 Rutgeerts i1). However, one female patient presented a severe endoscopic relapse in the left colon and a diffuse inflammation with a sub-stenosis in the anastomotic site (Figure 1). This patient presented a lymphoplasmacytic infiltration in the terminal ileum.

### 3.3. MRE Evaluation

All of the included patients underwent MRE evaluation after the first year of follow-up (median 18 months; range 13–52). The only patient that lost response to vedolizumab presented an increase in wall thickness and a contrast enhancement in the ileum, and the left colon at the MRE performed 19 months after the intestinal resection. This patient presented an ileocolic pattern ab initio (A3/L3/B3p).

**Figure 2:**
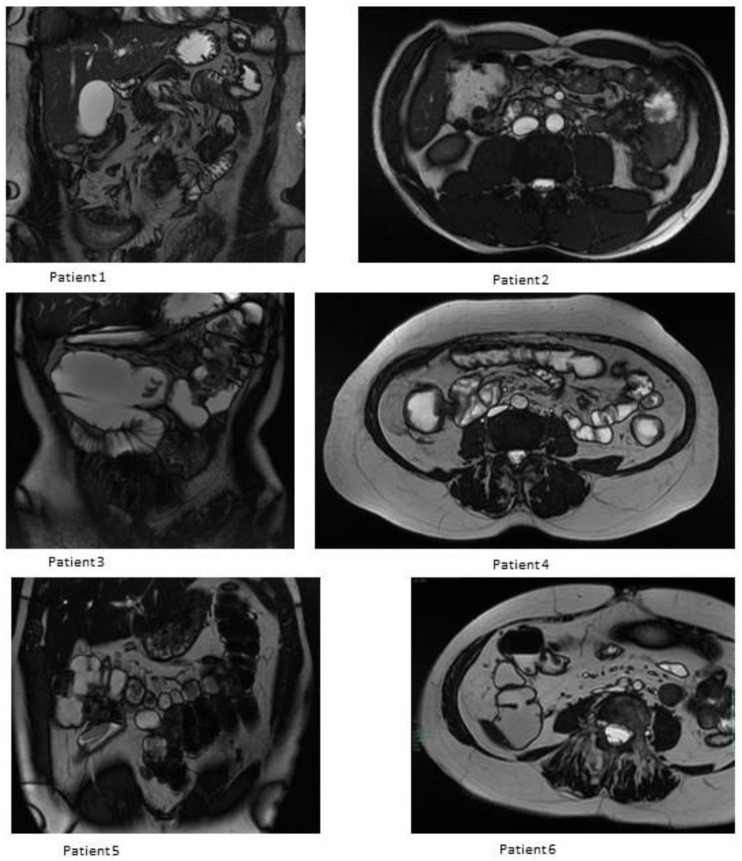
Anastomosis evaluation by magnetic resonance imaging after the first year of follow-up.

Only one other female patient presented a mild thickness in the ileum and sigmoid colon and a mild contrast enhancement in the same localizations after 52 months of follow-up (Figure 2).

Mild ileum contrast enhancement was observed in the other two patients after 13 and 29 months, respectively. None of the included patients presented fistula or stenosis.

## 4. Discussion

An etiological therapy for inflammatory bowel disease (IBD) does not yet exist. Anti-inflammatory, immunomodulators, monoclonal antibodies, and recently small molecules, have been used successfully since they can block the pathogenic path at one or more points of its complex course [3,26]. However, as this happens for all chronic inflammatory diseases, therapy is administered only after the inflammatory process has begun, after it has produced the first lesions, after symptoms have appeared, after they are interpreted and after the definitive diagnosis is made [3,27]. Thus, the treatment always arrives late. In general, the current ambitious management of IBD is focused on a treat-to-target strategy; that is, it attends to induce a deep remission with adjustment of appropriate medications according to well-defined treatment goals in both, CD and Ulcerative Colitis (UC) [21].

This strategy has overcome the historical IBD treatment strategy (focused on symptom resolution) as shown in Selecting Therapeutic Targets in IBD (STRIDE-2) recommendations [28].

Symptomatic relief and normalization of serum and fecal markers are considered short-term targets. Transmural healing in CD and histological healing in UC are desirable targets as surrogate markers of depth remission, however, there is currently no strong evidence concerning these two items [28,29]. Therefore, recently, the reduction or disappearance of lesions has become the main goal [28].

After intestinal resection for Crohn’s disease, we are faced with a unique experimental condition in which the mucosa, at least apparently, is completely healthy. However, we know in advance that it has a great probability of being attacked by a de novo inflammatory process, especially in high-risk patients. Anti-inflammatories, immunomodulators, antibiotics, and monoclonal antibodies have been used for the prevention of POR, but with poor results. A strategy including close surveillance and treatment of early lesions has also been proposed. However, in this study, all of these patients were followed up for 18 months [17].

In this paper, we report the results of preventive treatment with Vedolizumab, an anti-integrin drug that blocks lymphocytic trafficking in patients at high risk of recurrence after ileocecal resection. It is a case series extended for a long observational period (median: 51.5 range: 33–64 months) with strictly endoscopic and MRI evaluation. We chose Vedolizumab, since gut homing of T-cells is a crucial role in the pathogenesis of IBD [30]. Migration of effector T lymphocytes to the gut under antigenic attack contributes to the local production of pro-inflammatory mediators including IFN-γ, IL-6, IL-9, and IL-17 [31]. Thus, this process, although not the primum movens, represents an early moment of the inflammatory process and, therefore, theoretically, the most suitable pathogenetic moment to be blocked to prevent the amplification of inflammation [3].

In our series, one out of six patients (16%) presented a recurrence. It refers to the patient in whom there was also a colonic localization. It is interesting to note that after about a year of treatment, the patient had an exacerbation of the colic lesions and at that time the terminal neo ileum appeared completely normal. The stenosis of anastomosis was detected only at the follow-up after two years of treatment, completely asymptomatic. The other 5 patients were well, with an optimal quality of life, and maintain their remission without the need for drug escalation. These patients also refused the proposed de-escalation of therapy, fearing the bad experience of living with the active disease before the surgery.

Our study has some limitations. First, the inherent limits due to a non-randomized study of intervention. Second, the lack of a control group (ideally treated with another biological drug). Third, the lack of a radiological score to identify radiological recurrence.

Finally, the small sample size represents a non-negligible limit concerning the strength of the evidence. Therefore, larger postoperative controlled trials are needed to establish the effectiveness of Vedolizumab on the rate of POR. Only a few data are available concerning the use of Vedolizumab for the prevention and treatment of POR, and only anecdotal experience or retrospective studies concerning its application in preventing POR [14,15,16,32].

Recently, a prospective placebo-controlled randomized trial investigated the preventive effect of vedolizumab in CD patients following an ileocolonic resection (initiated within four weeks following the surgical resection).

The primary endpoint was the endoscopic POR rate at six months. Although a very short follow-up period, this RCT showed a lower incidence and severity of POR in vedolizumab-treated patients [33].

The two largest published experiences showed conflicting data. Yamada et al., in a retrospective study with a propensity-score matched analysis, showed lower rates of endoscopic remission (25 vs. 69%, *p* = 0.03) in patients treated with Vedolizumab as compared to anti-TNF-α agents. However, patients in the Vedolizumab group were more often younger, with a peri-anal disease, and presented multiple failures to the anti-TNFα therapy [14].

Another retrospective study evaluated the effectiveness of Vedolizumab for the treatment of postoperative recurrence (Rutgeerts ≥ 2 at colonoscopy performed 6–12 months after the surgical resection) in CD patients after ileocolic resection. Among 58 CD patients presenting a POR, Macaluso, and colleagues reported an endoscopic success (defined as a reduction of at least one point of Rutgeerts score) in 47.6% of patients. 19.0% and 32.8% of the included patients presented a clinical failure at one year, and at the end of follow-up, respectively (mean follow-up: 24.8 ± 13.1 months). Seven patients (12.1%) required a new surgical resection [32].

In a Real-Word multicenter European study including CD patients who underwent a curative ileocolonic resection who received early postoperative prophylaxis with a biologic agent, the endoscopic POR was observed in 41.8, 49 and 48.6% within 1, 2, and 3 years, respectively. In the vedolizumab and anti-TNFα groups, 33% and 40% of patients presented an endoscopic POR, respectively (*p* = 0.99) while 61% in the Ustekinumab group presented an endoscopic POR (*p* = 0.04).

In univariate analysis, the type of biologic prophylaxis treatment was associated with endoscopic POR at 1 year, (*p* = 0.031). Only past exposure to either infliximab or adalimumab and side-to-side and end-to-side anastomoses compared to end-to-end anastomosis were associated with the risk of endoscopic recurrence at one year. Thus, after controlling for disease severity characteristics, there were no differences in endoscopic POR risk between anti-TNF prophylaxis and the other treatment groups [16]. Moreover, another retrospective study including CD patients with a prevention strategy with Vedolizumab or Ustekinumab showed similar efficacy between the two therapies. The cumulative probability of clinical POR at 12 months after surgery was 32% and 30% for Ustekinumab and Vedolizumab, respectively [15].

The data deriving from our study showed an encouraging perspective, especially for the complex group at very high risk to develop a POR. The peculiarity of our experience is to introduce Vedolizumab as preventive therapy before the onset of new endoscopic lesions within 12 weeks after surgical resection. As a case series, we cannot conclude that the results of this study are only linked to the action of the drug, but this hypothesis cannot be completely rejected. Moreover, as suggested in a previously published study, the use of Vedolizumab in the postoperative setting could have another helpful effect in blocking the presence of submucosal lymphocytic plexytis in the proximal surgical margin [32]. Myenteric plexytis is a risk factor for POR after ileocolonic resection. Vedolizumab can block lymphocytic trafficking among myenteric and submucosal plexus, including those of the perioperative bowel [32,34,35,36].

These beneficial effects, in a context where the inflammatory burden has been markedly reduced by surgery, could represent the pathophysiological rationale of Vedolizumab efficacy and its usefulness as a prevention strategy of POR in high-risk CD patients.

## 5. Conclusions

These encouraging data suggest that early post-operative treatment with Vedolizumab may be a valuable strategy to be submitted to a prospective controlled trial for preventing POR.

## Figures and Tables

**Table 1:** Patients’ characteristics.

Sex Gender	Age at Diagnosis	Surgery-Free Survival (Years)	Indication to Surgery	Montreal Classification	Previous Medical Treatment	Previous Surgery	Age at Surgery	Weeks to VEDO Induction	First Colonoscopic Evaluation (Rutgeerts) (Months)	Second Colonoscopic Evaluation (Rutgeerts) (Months)	MRE (Months)	End of Follow-Up (Months)	Loss of Response (HBI)
Female	41	9	Inflammatory stenosis; Active penetrating disease	A3/L3/B3p	ADA	No	50	4	0 (10)	i4 (32)	T+: i, lc; E+: i, lc (19)	33	Yes (10)
Male	26	1	Inflammatory stenosis	A2/L3/B3	IFX	No	27	6	i1 (12)	i0(26)	T−; E+: i (13)	58	No (1)
Female	31	6 (1st resection) 16 (2nd resection)	Inflammatory stenosis; Active penetrating disease	A2/L3/B3p	IFX; AZA	Yes	57	10	0 (8)	0 (32)	T−; E− (17)	62	No (1)
Female	52	11	Inflammatory stenosis	A3/L1/B2	ADA	No	63	6	0 (12)	i0(33)	T+: lc, sc E+: lc (52)	64	No (1)
Male	53	2	Fibrotic stenosis	A3/L1/B2	IFX	No	54	12	0 (8)	i1 (25)	T−; E− (14)	45	No (4)
Female	62	2	Inflammatory stenosis;	A3/L3/B2	ADA	No	64	6	0 (8)	n.a.	T−; E+: i (29)	39	No (2)

VEDO: Vedolizumab; ADA: Adalimumab; IFX: Infliximab; AZA: Azathioprine; n.a.: Not available: thickness; E: iper-enhancement; i: ileum; rc: right colon; t: transverse; lc: left colon; sc: sigmoid colon; MRE: magnetic resonance enterography; HBI: Harvey-Bradshaw index.

**Table 2:** Risks factors of postoperative recurrence present in the 6 CD patients enrolled in the study.

Sex Gender	Perianal Localization	Active Smoking	Penetrating Disease at Index Surgery	Prior Intestinal Surgery	Granulomas	Myenteric Plexitis	Previous Loss of Response to Anti-TNFα
Female	yes	yes	No	no	n.a.	n.a.	yes
Male	no	yes	No	no	n.a.	n.a.	yes
Female	yes	no	No	yes	n.a.	n.a.	yes
Female	no	no	yes	no	n.a.	n.a.	yes
Male	no	no	yes	no	n.a.	n.a.	yes
Female	no	yes	No	no	n.a.	n.a.	yes

n.a. = not available.

## Data Availability

All data generated or analyzed during this study are included in this published article.
